# Progress in islet xenotransplantation: Immunologic barriers, advances in gene editing, and tolerance induction strategies for xenogeneic islets in pig-to-primate transplantation

**DOI:** 10.3389/frtra.2022.989811

**Published:** 2022-10-20

**Authors:** Daniel L. Eisenson, Yu Hisadome, Michelle R. Santillan, Kazuhiko Yamada

**Affiliations:** Department of Surgery, The Johns Hopkins Hospital, Baltimore, MD, United States

**Keywords:** xenotransplantation, transplantation tolerance, islet transplantation, porcine islet xenotransplantation, composite islet-kidney

## Abstract

Islet transplantation has emerged as a curative therapy for diabetes in select patients but remains rare due to shortage of suitable donor pancreases. Islet transplantation using porcine islets has long been proposed as a solution to this organ shortage. There have already been several small clinical trials using porcine islets in humans, but results have been mixed and further trials limited by calls for more rigorous pre-clinical data. Recent progress in heart and kidney xenograft transplant, including three studies of pig-to-human xenograft transplant, have recaptured popular imagination and renewed interest in clinical islet xenotransplantation. This review outlines immunologic barriers to islet transplantation, summarizes current strategies to overcome these barriers with a particular focus on approaches to induce tolerance, and describes an innovative strategy for treatment of diabetic nephropathy with composite islet-kidney transplantation.

## Introduction

Nearly one in ten Americans suffers from diabetes mellitus. Unfortunately, incidence is increasing in the United States and across the globe (1, 2). Diabetes is a leading cause of both cardiovascular disease and end stage renal disease (ESRD) (3, 4), and carries particularly high short-term mortality in patients with hypoglycemic unawareness (5). Human islet transplantation has emerged as one effective treatment for diabetic patients with hypoglycemic unawareness (6), but this procedure is rare due, in part, to a shortage of deceased donor pancreases (7). Xenotransplantation using islets obtained from pigs may overcome this organ shortage and allow for broader application of islet transplantation.

There have already been several small clinical studies using porcine islets in humans (8–10), but results have been mixed and further trials limited by more recent consensus guidelines calling for more rigorous pre-clinical data (11). However, recent studies in pig-to-human heart and kidney xenograft transplantation have reignited public enthusiasm for xenotransplantation and may reopen debate about when to initiate clinical trials in islet xenograft transplantation (12–14). This review describes the current status of xeno islet transplantation, outlining immunologic barriers to islet transplantation, current strategies to overcome these barriers including genetic modification and novel immunosuppression, and innovative strategies to induce tolerance in xenotransplantation.

### Immunologic barriers to islet xenotransplantation

Pig islets, like other porcine xenografts, precipitate vigorous immune responses in humans involving both innate and adaptive immune components (15). Innate barriers in pig-to-human transplantation include preformed natural antibodies to carbohydrate antigens on porcine endothelial cells, complement and coagulation system dysregulation due to species incompatibilities, and activation of macrophages and natural killer cells (16). In auto- and allotransplantation models of islet transplantation, islets injected into the portal vein trigger an immediate inflammatory response, known as instant blood-mediated inflammatory reaction (IBMIR) (17, 18). While the exact mechanism is unknown, IBMIR is believed to be triggered by tissue factor expressed on islets, which leads to activation of innate immune components including complement and coagulation systems and subsequent loss of islets (19, 20). In islet xenotransplantation, preformed antibody-binding and complement/coagulation dysregulation contribute to more robust IBMIR with greater islet losses (21, 22) as high as 70% in some studies (23).

The adaptive immune system represents another major barrier to the long-term viability of xenografts. Although it was initially believed that species incompatibilities between human leukocyte antigen (HLA) and swine leukocyte antigen (SLA) proteins would inhibit effective T cell activation as was seen in pig-to-mouse transplant models, *in vitro* and *in vivo* studies have since demonstrated that T cells are *directly* activated by SLA-TCR binding (24) and costimulatory interactions are not limited by the same pig-to-mouse species incompatibilities (25). T and B cells are also *indirectly* activated by pig antigens, leading to recruitment of macrophages and NK cells, and elicited antibody production (26, 27). Xenogeneic islets may be particularly vulnerable to T cell mediated rejection as studies have shown that prolonged survival of porcine xenografts in athymic mice (28) and prolonged islet survival using T cell targeted immunosuppression strategies (29). However, as is true in all pig-to-primate transplants, humoral immunity remains a critical obstacle to xenograft survival (30).

### Strategies to overcome immune barriers: Encapsulation, genetic modification, and novel immunosuppression

Given these greater immunologic hurdles, several strategies have been developed and studied to prolong xenogeneic islet survival. These include innovative delivery systems with micro-and macro-encapsulation of islets, genetic modification of donor pigs to reduce immunogenicity, and novel immunosuppression using costimulatory blockade. Additionally, while portal vein infusion is the preferred injection site in human islet transplantation, injection sites are more varied in xenogeneic islet transplantation studies. There are not enough comparative studies to draw any conclusions about which injection site is superior, but we will discuss possible advantages of one site (renal subcapsular injection) in more detail later in this review (31, 32).

#### Innovative delivery systems: Islet encapsulation

Free islet transplantation is standard of care in allogeneic islet transplantation, but immunosuppression is required to achieve modest success in humans—which is associated with increased risks of infectious disease and malignancy—and results remain substandard in pig-to-primate xenotransplantation. Several groups have developed encapsulation technologies to protect islets from immune and inflammatory responses while allowing for uptake of nutrients and release of insulin. Proponents of encapsulation strategies argue that these technologies may prolong xenograft survival without immunosuppression. In broad strokes, these strategies include microencapsulation of islets in alginate matrix, and macro-encapsulation of immobilized islets in bilayered PTFE with a common oxygenation chamber (33). Encapsulated islets are subsequently transplanted in the peritoneal cavity. There have been nationally regulated clinical studies of microencapsulated islets in New Zealand and in Argentina, which confirmed the safety of this technique but failed to demonstrate meaningful clinical impact (34, 35). Macroencapsulation involves utilization of implantable devices with immobilized islets sharing a common oxygenated chamber. These technologies are less studied but there are some encouraging pre-clinical results, including pig-to-primate study in which microencapsulated islets reversed diabetes for 6 months in diabetic NHPs (36–38).

#### Genetic modification of pig donors

As mentioned earlier, innate immune barriers in pig-to-primate transplantation include preformed natural antibodies (Nabs) to porcine carbohydrate antigens as well as regulatory protein species incompatibilities that lead to dysregulation of complement and coagulation systems. In solid organ xenotransplantation, preformed antibody binding leads to hyperacute rejection of graft; in free islet xenotransplantation, preformed antibody binding leads to an amplified IBMIR with massive islet loss.

The most prevalent natural antibody targets the carbohydrate component (α−1,3-galactose, or “α-gal”) of a cell surface glycoprotein produced by an enzyme (α- 1,3-galactosyltransferase) that is not functional in humans or old-world primates (39). Accordingly, the creation of α-gal knockout (GalTKO) pigs represented a major breakthrough in the field of pig-to-primate transplantation (40–42), with improved xenograft survival in pig-to-primate heart and kidney pre-clinical studies (43–45). The impact of using GalTKO source pigs on xenograft survival in islet transplantation is less conclusive, which may be a function of changes in α-gal expression with islet maturation (46): studies using GalTKO adult pig islets demonstrate no benefit as compared to wild type adult pig islets (47), whereas studies using GalTKO neonatal islet cell clusters show increased rates of insulin independence (48). Other natural antibody targets include Neu5Gc made by the cytidine monophosphate-N-acetylneuraminic acid hydroxylase (CMAH) enzyme, and the blood group antigen SDa, made by β-1,4-N-acetyl-galactosaminyl transferase 2 (β4GALNT2) (49, 50). Cells from triple knockout (Gal, CMAH, and β4GALNT2 deficient) demonstrate reduced human antibody binding in vitro, but more studies are needed to determine whether elimination of all three genes is necessary in pig-to-primate solid organ and islet transplantation (51, 52).

Species incompatibilities between pig and human complement regulatory proteins pose another innate immune barrier to xenograft survival. These incompatibilities and the ensuing complement dysregulation may be circumvented through creation of “transgenic” pigs expressing human complement regulatory proteins, including hCD46, hCD55, and hCD59. Although expression of individual complement regulatory transgenes may not significantly reduce incidence of IBMIR (47), xenograft survival may be improved when combining carbohydrate antigen gene knockouts with complement regulatory transgenes: xenogeneic islets from multiply modified pigs (GalTKO + hCD55 + hCD59 or GalTKO + hCD39 + hCD46) demonstrated attenuated IBMIR and reduced islet loss (53, 54), and more recently, improved islet function and survival with insulin independence >1 year in a pig-to-baboon pre-clinical model (55). Although creation of multiply genetically modified pigs was challenging and prohibitively slow 20 years ago, the recent development of rapid gene editing tools including CRISPR-Cas9 has transformed production and availability of these multiply modified animals.

#### Novel immunosuppression

Advances in genetic engineering have enabled researchers to overcome key innate immune barriers in pig-to-primate transplantation, but vigorous B and T cell responses limit the long-term viability of xenografts. Standard immunosuppression regimens used in allotransplantation do not work in solid organ pig-to-primate xenotransplantation (56), as humoral immunity, which is difficult to control with standard immunosuppression regimens, plays a dominant role in rejection of xenografts. The CD40-CD154 pathway influences T cell-dependent antibody production, and modulation of this costimulatory interaction seems to be critical for rejection-free survival in heart and kidney xenograft transplantation (45, 56–58). Costimulation blockade has also proven important in pig-to-primate islet transplantation: immunosuppressive regimens including blockade of CD40-CD154 pathway are associated with prolonged islet survival and insulin independence in diabetic recipients (29, 55, 59, 60).

Unfortunately, anti-CD154 may be thrombogenic, raising concerns about the clinical applicability of these immunosuppression regimens (11, 61–63). Given these concerns, other groups have tried to replicate earlier results with regimens that do not include anti-CD154. Shin et al found that regimens replacing anti-CD154 with both tacrolimus and anti-CD40 (210R4) prolonged islet survival, although these regimens remained inferior to those containing anti-CD154 (64, 65). Higher doses of anti-CD40mAb may be needed to achieve the same results, as suggested by results from heart xenotransplantation studies (66). Another group has demonstrated prolonged islet survival with regimens targeting lymphocyte function-associated antigen 1 (LFA-1) instead of costimulation blockade (67). Although encouraging, additional studies using anti-LFA-1-based immunosuppression regimens are needed.

### Strategies to overcome immune barriers: Tolerance induction

Despite remarkable progress with gene editing and new immunosuppression protocols, these studies suggest that the adaptive immune responses—particularly antibody-mediated rejection—continue to limit the long-term viability of these transplants. Accordingly, strategies to induce tolerance may be a necessary adjunct to immunosuppression in clinical xenotransplantation. The tolerance strategies discussed here include regulatory T cell (Treg) infusions, mixed hematopoietic cell chimerism, and thymus transplantation.

#### Induction of tolerance with Tregs in islet transplantation

Regulatory T cells (Tregs) have been shown to play a key role in preventing rejection of transplanted allografts (68) *via* prevention of effector T cell responses and even inhibition of innate immune responses (69). This organic peripheral tolerance, mediated by host Tregs, has given rise to a targeted strategy to induce tolerance with transfer of *in vitro* culture expanded Tregs (70). Recipient culture expanded Tregs, also called autologous polyclonal Tregs, have been shown to promote tolerance of co-transplanted islets across allogeneic barriers in mice and are under active investigation in clinical allogeneic islet transplant trials in humans (NCT03444064) (71).

Autologous polyclonal Tregs may also promote tolerance across xenogeneic barriers. Treg infusions have been shown to delay porcine islet xenograft rejection in mouse models through control of effector T cell function (72). However, results of large animal islet xenograft transplantation with autologous Tregs have been mixed: although some animals have been able to achieve insulin independence while on immunosuppression, cessation of immunosuppression leads to complete rejection of islet grafts (73). To date, pre-clinical and clinical studies of culture expanded Tregs in allogeneic and xenogeneic islet transplantation have failed to show durable tolerance and have failed to consistently achieve insulin independence. Still, autologous polyclonal Tregs remain attractive to investigators given their safety profile and promising data from mouse experiments: one of the only ongoing clinical trials in porcine islet xenotransplantation uses autologous polyclonal Tregs in patients with Type 1 DM (NCT03162237) (71).

#### Mixed hematopoietic cell chimerism

**FIGURE 1:**
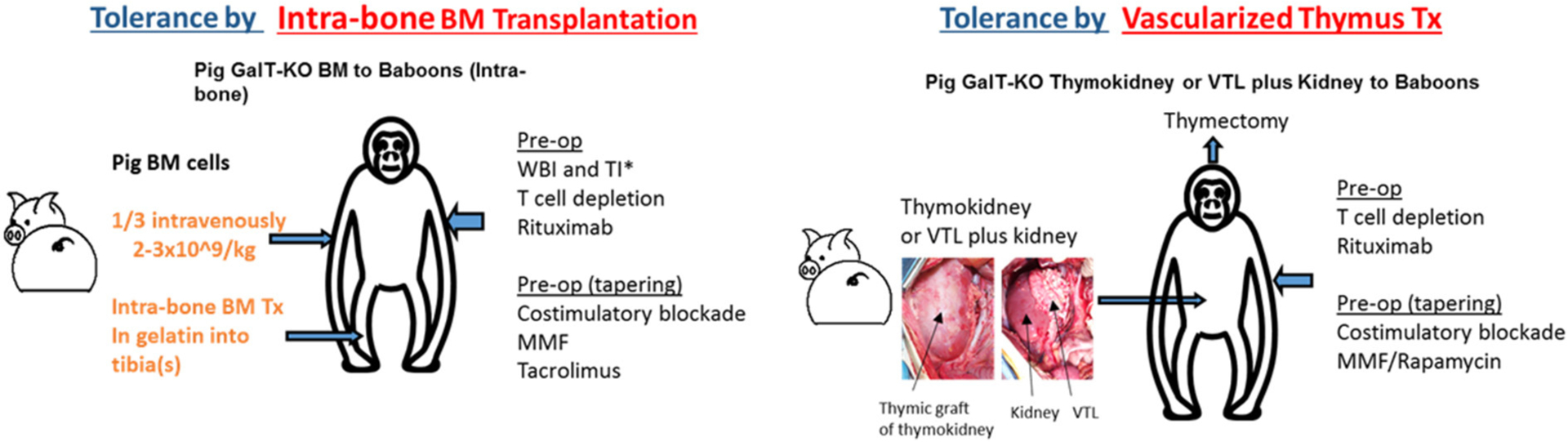
Two major strategies for the induction of tolerance of xenograft in the author’s laboratory: (1) Intra-bone bone marrow transplantation followed by solid organ transplantation, (2) Co-transplantation of vascularized thymic grafts with solid organs.

Another strategy to induce donor-specific immunologic tolerance involves combined organ and hematopoietic stem cell transplantation (HSCTx) from the same donor. When HSCTx is performed after non-myeloablative conditioning, donor hematopoietic cells coexist with recipient cells, resulting in “mixed” chimerism—in contrast to “full” chimerism, seen after myeloablative conditioning, where no hematopoietic cells of recipient origin are present and recipients may be at risk for immune-incompetence (74). This strategy has been used to achieve tolerance of allogeneic kidneys in multiple clinical studies (75–77), and has been shown to promote survival of allogeneic islets after withdrawal of immunosuppression in NHP pre-clinical models (78). However, it has proven more challenging to reproduce these results and establish mixed chimerism across xenogeneic barriers. Early studies demonstrated successful tolerance induction in pig-to-mouse transplantation models (79, 80), but xenogeneic cells are rapidly eliminated after HSCTx in pig-to-primate transplantation models (81, 82). There has been recent progress in this area. The insertion of a human macrophage inhibitory protein, hCD47, prevents phagocytosis of porcine hematopoietic stem cells and prolongs both chimerism and donor skin grafts in a pig-to-baboon skin transplant model after HSCTx (83). Additionally, injection of porcine hematopoietic stem cells directly into recipient bone marrow has also been shown to prolong chimerism and even achieve bone marrow engraftment in recipient NHPs (Figure 1) (84, 85). Combining these techniques may have synergistic effects, but chimerism is lost by 60 days after HSCTx. More work is needed to reliably establish mixed chimerism across xenogeneic barriers in large animal studies before this strategy can be applied to islet xenografts.

#### Vascularized thymic graft transplantation

Although less studied in clinical transplantation, vascularized thymic graft transplantation has been shown to be a powerful strategy to induce tolerance in pre-clinical models. The thymus plays a critical role in deletion of autoreactive T cells, and so it was hypothesized that thymic tissue transplantation may promote tolerance *via* central deletion of donor-reactive T cells. This theory was validated in mouse models across allogeneic barriers, and subsequently validated in pig-to-mouse studies which demonstrated that transplantation of devascularized porcine thymic tissue enabled tolerance of porcine skin grafts *via* central deletion of pig-reactive T cells (86). However, devascularized thymic tissue was rapidly rejected in large animal models. To avert this prompt destruction of implanted thymic tissue before it was able to participate in tolerance induction, techniques were developed to transplant *vascularized* thymic tissue. These included (1) pre-vascularization of thymic tissue underneath donor kidney capsule with subsequent transplantation of composite thymus and kidney (“thymokidney”), and (2) tracing diminutive thymic vessels to their larger originating vessels, and transplanting thymic tissue with larger source vessels as a vascularized thymic lobe (Figure 1) (87, 88). Unlike previous attempts to induce tolerance in large animals using devascularized thymic tissue, these vascularized thymic grafts facilitated donor-specific tolerance across allogeneic and xenogeneic barriers (89–91). When GalTKO pigs first became available, the addition of vascularized thymic grafts to GalTKO pig-to-NHP kidney transplant extended kidney xenograft survival from 29 to 83 days (43). Combining vascularized thymic graft transplantation with novel immunosuppression including costimulatory blockade and CTLA4-Ig has yielded further improvements, with survivals now reliably >6 months using GalTKO pigs without additional genetic modifications (45).

Vascularized thymic graft co-transplantation has been demonstrated to be one of the most effective strategies to induce tolerance and prolong xenograft survival in pre-clinical pig-to-NHP studies and may be a key component of the first clinical trials in pig-to-human kidney transplantation. Indeed, this tolerance strategy was employed in two highly publicized pig-to-human kidney transplants in brain dead patients at New York University in 2021 (NYTimes, October 21st, 2021).

One of the challenges with islet transplantation for diabetes is that the morbidity associated with immunosuppression already limits the potential recipients: current data only supports human islet transplantation or pancreas transplantation for specific indications including hypoglycemic unawareness or concurrent renal disease. Accordingly, despite encouraging results with pig-to-NHP vascularized thymic grafts co-transplanted along with kidneys, thymic graft transplantation is invasive and may be difficult to justify in conjunction with porcine islets in patients with diabetes alone. As we will discuss in the next section, diabetic nephropathy presents a specific—and growing—indication for a new technique designed to combine islet-preservation and tolerance strategies.

## Cure for diabetic nephropathy using composite islet-kidney + vascularized thymic lobe transplantation

**FIGURE 2:**
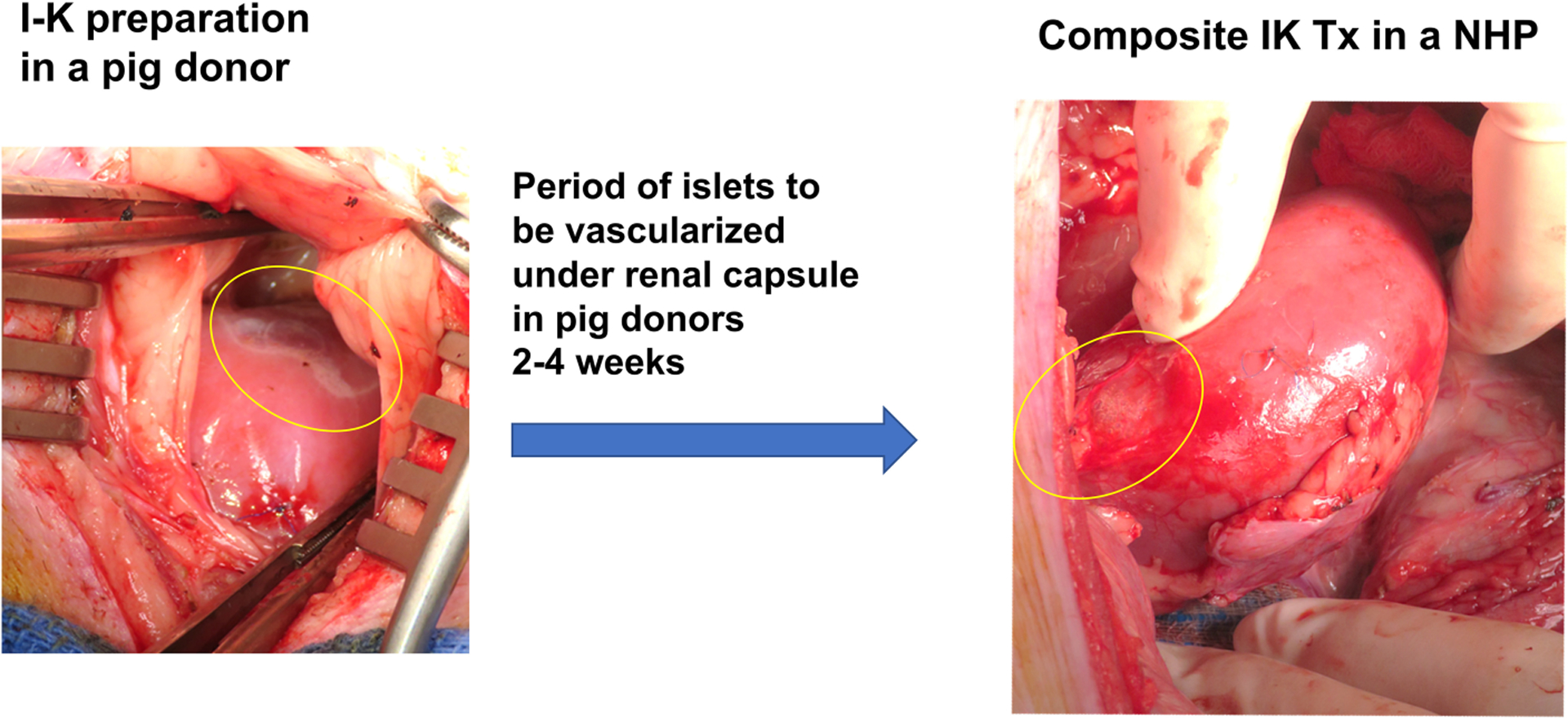
Composite Islet-Kidney preparation and transplantation.

Diabetes is a major cause of ESRD and is a risk factor for perioperative mortality and premature graft loss after kidney transplantation. The senior author of this review developed a strategy to cure both diabetes and end stage renal disease with transplantation of porcine composite islet-kidney along with vascularized thymic graft (Figure 2) (88–90). As detailed above, xenogeneic islets are susceptible to destruction by both innate and adaptive mechanisms. This strategy counters innate immune destruction of islets by circumventing the typical pathway that triggers IBMIR, which is amplified in xenogeneic islet transplantation, and takes advantage of the relative immune privilege of the kidney. Porcine islets are isolated and *pre-vascularized* under autologous renal capsule, with subsequent transplantation of composite islet-kidney (92). Preclinical allotransplantation studies in pigs and in NHPs have demonstrated that this procedure preserves islets, likely by limiting innate immune destruction: diabetes is cured in animals who undergo composite islet kidney transplantation, while animals who undergo conventional free islet injection with the same islet equivalents (IEQs) remain insulin dependent (93, 94). Combined islet-kidney transplantation for patients with diabetic nephropathy also allows for co-transplantation of vascularized thymic grafts, which is one of the most promising tolerance induction strategies in pig-to-NHP kidney xenograft transplantation and may effectively counter B and T cell-mediated islet destruction.

On a practical level, this strategy tilts the risk-benefit calculus in favor of islet transplantation. Patients receiving kidney graft have already committed to lifelong immunosuppression, and so concerns about long-term consequences of immunosuppression are not relevant when considering co-transplantation of islets. Similarly, patients receiving kidney graft have already committed to an operation, and so may receive vascularized thymic grafts without need for an additional invasive procedure. Lastly, islets transplanted in islet-kidney grafts are localized and easy to remove with the kidney graft in the event of rejection, whereas free islets injected into the portal vein are diffusely engrafted throughout the liver and not amenable to removal. Ongoing research will confirm whether these approaches are specifically effective for pig-to-NHP islet transplantation, but combined islet-kidney xenograft transplantation for diabetic nephropathy is a promising strategy for a targeted population.

## Conclusion

Clinical islet transplantation is limited by a relatively static supply of donor pancreases. Porcine islet xenotransplantation has long been proposed as a solution to this organ shortage, but enthusiasm for islet xenotransplantation waned in the face of mixed results from pre-clinical and early clinical studies. However, progress in the field of kidney and heart xenotransplantation with the development of rapid genome editing technologies, novel immunosuppression regimens, and even tolerance induction strategies has led to significant improvements in pig-to-NHP heart and kidney graft survival in recent years. As described in this review, these broader developments in xenotransplantation have contributed to specific improvements in pig-to-NHP islet xenograft transplantation, with recent studies demonstrating reversal of diabetes with costimulation-based immunosuppression protocols using islets obtained from multiply modified pigs (55).

Recent pig-to-human heart and kidney xenograft transplants have recaptured the popular imagination. As the transplant community nears clinical trials in pig-to-human heart and kidney xenograft transplantation (https://www.wsj.com/articles/fda-said-to-plan-pig-organ-transplant-clinical-trials-11656622411), there is renewed interest in clinical islet xenotransplantation. Given recent progress, there may be adequate pre-clinical efficacy and safety data to initiate select clinical trials within targeted patient populations, according to guidelines established by the International Xenotransplantation Association (11). The results of ongoing pre-clinical studies focusing on further genetic modifications of donor pigs, optimizing costimulation blockade without anti-CD154 mAb, and applying tolerance-inducing strategies to pre-clinical islet xenotransplantation will provide more clarity on the question of when islet xenotransplantation will become a viable clinical solution to diabetes.

## Abbreviations:

β4GALNT2: β−1,4-N-acetyl-galactosaminyltransferase
CMAH: cytidine-monophosphate-N-acetyl-neuraminic acid hydroxylase
GalTKO: α1,3 Galactosyl transferase gene knockout
hCD55: human CD55
hCD59: human CD59
hCD47: human CD47
HLA: human leukocyte antigen
IBBMTx: intra-bone bone marrow transplantation
IBMIR: instant blood-mediated inflammatory reaction
IK: islet-kidney
HSCTx: hematopoietic stem cell transplantation
LFA-1: lymphocyte function-associated antigen 1
mAb: monoclonal antibody
Nab: natural antibody
NHP: non-human primate
NK: natural killer
NYU: New York University
PTFE: PolyTetraFluoroEthylene
SLA: swine leukocyte antigen
Treg: regulatory T cells

## References

[1] World Health Organization. Global Report on Diabetes. World Health Organization (?2016)?. Available online at: https://apps.who.int/iris/handle/10665/204871.

[2] Centers for Disease Control and Prevention. National Diabetes Statistics Report. Centers for Disease Control and Prevention. Available online at: https://www.cdc.gov/diabetes/data/statistics-report/index.html.

[3] Emerging Risk Factors Collaboration, Sarwar N, Gao P, Seshasai SR, Gobin R, Kaptoge S, Diabetes mellitus, fasting blood glucose concentration, and risk of vascular disease: a collaborative meta-analysis of 102 prospective studies. Lancet. (2010) 375:2215–22. doi: 10.1016/S0140-6736(10)60484-9 20609967 PMC2904878

[4] Saran R, Li Y, Robinson B, Ayanian J, Balkrishnan R, Bragg-Gresham J, US renal data system 2014 annual data report: epidemiology of kidney disease in the United States. Am J Kidney Dis. (2015) 66 (1 Suppl. 1):Svii, S1–305. doi: 10.1053/j.ajkd.2015.05.001 PMC6643986 26111994

[5] McCoy RG, Van Houten HK, Ziegenfuss JY, Shah ND, Wermers RA, Smith SA. Increased mortality of patients with diabetes reporting severe hypoglycemia. Diabetes Care. (2012) 35:1897–901. doi: 10.2337/dc11-2054 22699297 PMC3425008

[6] Shapiro AM, Pokrywczynska M, Ricordi C. Clinical pancreatic islet transplantation. Nat Rev Endocrinol. (2017) 13:268–77. doi: 10.1038/nrendo.2016.178 27834384

[7] Gamble A, Pepper AR, Bruni A, Shapiro AMJ. The journey of islet cell transplantation and future development. Islets. (2018) 10:80–94. doi: 10.1080/19382014.2018.1428511 29394145 PMC5895174

[8] Groth CG, Korsgren O, Tibell A, Tollemar J, Moller E, Bolinder J, Transplantation of porcine fetal pancreas to diabetic patients. Lancet. (1994) 344:1402–4. doi: 10.1016/S0140-6736(94)90570-3 7968077

[9] Valdes-Gonzalez RA, Dorantes LM, Garibay GN, Bracho-Blanchet E, Mendez AJ, Davila-Perez R, Xenotransplantation of porcine neonatal islets of langerhans and sertoli cells: a 4-year study. Eur J Endocrinol. (2005) 153:419–27. doi: 10.1530/eje.1.01982 16131605

[10] Valdes-Gonzalez R, Rodriguez-Ventura AL, White DJ, Bracho-Blanchet E, Castillo A, Ramirez-Gonzalez B, Long-term follow-up of patients with type 1 diabetes transplanted with neonatal pig islets. Clin Exp Immunol. (2010) 162:537–42. doi: 10.1111/j.1365-2249.2010.04273.x 20964645 PMC3026557

[11] Hering BJ, Cozzi E, Spizzo T, Cowan PJ, Rayat GR, Cooper DK, First update of the international xenotransplantation association consensus statement on conditions for undertaking clinical trials of porcine islet products in type 1 diabetes—Executive summary. Xenotransplantation. (2016) 23:3–13. doi: 10.1111/xen.12231 26940725

[12] Griffith BP, Goerlich CE, Singh AK, Rothblatt M, Lau CL, Shah A, Genetically modified porcine-to-human cardiac xenotransplantation. N Engl J Med. (2022) 387:35–44. doi: 10.1056/NEJMoa2201422 35731912 PMC10361070

[13] Porrett PM, Orandi BJ, Kumar V, Houp J, Anderson D, Cozette Killian A, First clinical-grade porcine kidney xenotransplant using a human decedent model. Am J Transplant. (2022) 22:1037–53. doi: 10.1111/ajt.16930 35049121

[14] Montgomery RA, Stern JM, Lonze BE, Tatapudi VS, Mangiola M, Wu M, Results of two cases of pig-to-human kidney xenotransplantation. N Engl J Med. (2022) 386:1889–98. doi: 10.1056/NEJMoa2120238 35584156

[15] Mok D, Black M, Gupta N, Arefanian H, Tredget E, Rayat GR. Early immune mechanisms of neonatal porcine islet xenograft rejection. Xenotransplantation. (2019) 26:e12546. doi: 10.1111/xen.12546 31410915

[16] Eisenson DL, Hisadome Y, Yamada K. Progress in xenotransplantation: immunologic barriers, advances in gene editing, and successful tolerance induction strategies in pig-to-primate transplantation. Front Immunol. (2022) 13:899657. doi: 10.3389/fimmu.2022.899657 35663933 PMC9157571

[17] Naziruddin B, Iwahashi S, Kanak MA, Takita M, Itoh T, Levy MF. Evidence for instant blood-mediated inflammatory reaction in clinical autologous islet transplantation. Am J Transplant. (2014) 14:428–37. doi: 10.1111/ajt.12558 24447621

[18] Nilsson B, Ekdahl KN, Korsgren O. Control of instant blood-mediated inflammatory reaction to improve islets of langerhans engraftment. Curr Opin Organ Transplant. (2011) 16:620–6. doi: 10.1097/MOT.0b013e32834c2393 21971510

[19] Ji M, Yi S, Smith-Hurst H, Phillips P, Wu J, Hawthorne W, The importance of tissue factor expression by porcine NICC in triggering IBMIR in the xenograft setting. Transplantation. (2011) 91:841–6. doi: 10.1097/TP.0b013e3182106091 21325994

[20] Kourtzelis I, Magnusson PU, Kotlabova K, Lambris JD, Chavakis T. Regulation of instant blood mediated inflammatory reaction (IBMIR) in pancreatic islet xeno-transplantation: points for therapeutic interventions. Adv Exp Med Biol. (2015) 865:171–88. doi: 10.1007/978-3-319-18603-0_11 26306450

[21] Bennet W, Sundberg B, Lundgren T, Tibell A, Groth CG, Richards A, Damage to porcine islets of langerhans after exposure to human blood *in vitro*, or after intraportal transplantation to cynomologus monkeys: protective effects of sCR1 and heparin. Transplantation. (2000) 69:711–9. doi: 10.1097/00007890-200003150-00007 10755515

[22] Miyagawa S, Yamamoto A, Matsunami K, Wang D, Takama Y, Ueno T, Complement regulation in the GalT KO era. Xenotransplantation. (2010) 17:11–25. doi: 10.1111/j.1399-3089.2010.00569.x 20149185

[23] Davalli AM, Ogawa Y, Scaglia L, Wu YJ, Hollister J, Bonner-Weir S, Function, mass, and replication of porcine and rat islets transplanted into diabetic nude mice. Diabetes. (1995) 44:104–11. doi: 10.2337/diab.44.1.104 7813803

[24] Yamada K, Sachs DH, DerSimonian H. Human anti-porcine xenogeneic T cell response. Evidence for allelic specificity of mixed leukocyte reaction and for both direct and indirect pathways of recognition. J Immunol. (1995) 155:5249–56. 7594537

[25] Kalscheuer H, Onoe T, Dahmani A, Li HW, Holzl M, Yamada K, Xenograft tolerance and immune function of human T cells developing in pig thymus xenografts. J Immunol. (2014) 192:3442–50. doi: 10.4049/jimmunol.1302886 24591363 PMC3983999

[26] Dorling A, Lombardi G, Binns R, Lechler RI. Detection of primary direct and indirect human anti-porcine T cell responses using a porcine dendritic cell population. Eur J Immunol. (1996) 26:1378–87. doi: 10.1002/eji.1830260630 8647220

[27] Galili U Induced anti-non gal antibodies in human xenograft recipients. Transplantation. (2012) 93:11–6. doi: 10.1097/TP.0b013e31823be870 22146315

[28] Karlsson-Parra A, Ridderstad A, Wallgren AC, Moller E, Ljunggren HG, Korsgren O. Xenograft rejection of porcine islet-like cell clusters in normal and natural killer cell-depleted mice. Transplantation. (1996) 61:1313–20. doi: 10.1097/00007890-199605150-00005 8629289

[29] Hering BJ, Wijkstrom M, Graham ML, Hardstedt M, Aasheim TC, Jie T, Prolonged diabetes reversal after intraportal xenotransplantation of wild-type porcine islets in immunosuppressed nonhuman primates. Nat Med. (2006) 12:301–3. doi: 10.1038/nm1369 16491083

[30] Vadori M, Cozzi E. The immunological barriers to xenotransplantation. Tissue Antigens. (2015) 86:239–53. doi: 10.1111/tan.12669 26381044

[31] Kim HI, Yu JE, Park CG, Kim SJ. Comparison of four pancreatic islet implantation sites. J Korean Med Sci. (2010) 25:203–10. doi: 10.3346/jkms.2010.25.2.203 20119571 PMC2811285

[32] Jindal RM, Sidner RA, McDaniel HB, Johnson MS, Fineberg SE. Intraportal vs kidney subcapsular site for human pancreatic islet transplantation. Transplant Proc. (1998) 30:398–9. doi: 10.1016/S0041-1345(97)01327-4 9532100

[33] Liu Z, Hu W, He T, Dai Y, Hara H, Bottino R, Pig-to-Primate islet xenotransplantation: past, present, and future. Cell Transplant. (2017) 26:925–47. doi: 10.3727/096368917X694859 28155815 PMC5657750

[34] Wynyard S, Nathu D, Garkavenko O, Denner J, Elliott R. Microbiological safety of the first clinical pig islet xenotransplantation trial in New Zealand. Xenotransplantation. (2014) 21:309–23. doi: 10.1111/xen.12102 24801820

[35] Matsumoto S, Abalovich A, Wechsler C, Wynyard S, Elliott RB. Clinical benefit of islet xenotransplantation for the treatment of type 1 diabetes. EBioMedicine. (2016) 12:255–62. doi: 10.1016/j.ebiom.2016.08.034 27592597 PMC5078586

[36] Dufrane D, Goebbels RM, Gianello P. Alginate macroencapsulation of pig islets allows correction of streptozotocin-induced diabetes in primates up to 6 months without immunosuppression. Transplantation. (2010) 90:1054–62. doi: 10.1097/TP.0b013e3181f6e267 20975626

[37] Ludwig B, Rotem A, Schmid J, Weir GC, Colton CK, Brendel MD, Improvement of islet function in a bioartificial pancreas by enhanced oxygen supply and growth hormone releasing hormone agonist. Proc Natl Acad Sci USA. (2012) 109:5022–7. doi: 10.1073/pnas.1201868109 22393012 PMC3324017

[38] Ludwig B, Reichel A, Steffen A, Zimerman B, Schally AV, Block NL, Transplantation of human islets without immunosuppression. Proc Natl Acad Sci USA. (2013) 110:19054–8. doi: 10.1073/pnas.1317561110 24167261 PMC3839710

[39] Galili U, Rachmilewitz EA, Peleg A, Flechner I. A unique natural human IgG antibody with anti-alpha-galactosyl specificity. J Exp Med. (1984) 160:1519–31. doi: 10.1084/jem.160.5.1519 6491603 PMC2187506

[40] Kolber-Simonds D, Lai L, Watt SR, Denaro M, Arn S, Augenstein ML, Production of alpha-1,3-galactosyltransferase null pigs by means of nuclear transfer with fibroblasts bearing loss of heterozygosity mutations. Proc Natl Acad Sci USA. (2004) 101:7335–40. doi: 10.1073/pnas.0307819101 15123792 PMC409919

[41] Lai L, Kolber-Simonds D, Park KW, Cheong HT, Greenstein JL, Im GS, Production of alpha-1,3-galactosyltransferase knockout pigs by nuclear transfer cloning. Science. (2002) 295:1089–92. doi: 10.1126/science.1068228 11778012

[42] Dai Y, Vaught TD, Boone J, Chen SH, Phelps CJ, Ball S, Targeted disruption of the alpha1,3-galactosyltransferase gene in cloned pigs. Nat Biotechnol. (2002) 20:251–5. doi: 10.1038/nbt0302-251 11875425

[43] Yamada K, Yazawa K, Shimizu A, Iwanaga T, Hisashi Y, Nuhn M, Marked prolongation of porcine renal xenograft survival in baboons through the use of alpha1,3-galactosyltransferase gene-knockout donors and the cotransplantation of vascularized thymic tissue. Nat Med. (2005) 11:32–4. doi: 10.1038/nm1172 15619627

[44] Kuwaki K, Tseng YL, Dor FJ, Shimizu A, Houser SL, Sanderson TM, Heart transplantation in baboons using alpha1,3-galactosyltransferase gene-knockout pigs as donors: initial experience. Nat Med. (2005) 11:29–31. doi: 10.1038/nm1171 15619628

[45] Rivard CJ, Tanabe T, Lanaspa MA, Watanabe H, Nomura S, Andres-Hernando A, Upregulation of CD80 on glomerular podocytes plays an important role in development of proteinuria following pig-to-baboon xeno-renal transplantation - an experimental study. Transpl Int. (2018) 31:1164–77. doi: 10.1111/tri.13273 29722117 PMC6407427

[46] Rayat GR, Rajotte RV, Hering BJ, Binette TM, Korbutt GS. In vitro and in vivo expression of Galalpha-(1,3)Gal on porcine islet cells is age dependent. J Endocrinol. (2003) 177:127–35. doi: 10.1677/joe.0.1770127 12697044

[47] van der Windt DJ, Bottino R, Casu A, Campanile N, Smetanka C, He J, Long-term controlled normoglycemia in diabetic non-human primates after transplantation with hCD46 transgenic porcine islets. Am J Transplant. (2009) 9:2716–26. doi: 10.1111/j.1600-6143.2009.02850.x 19845582

[48] Thompson P, Badell IR, Lowe M, Cano J, Song M, Leopardi F, Islet xenotransplantation using gal-deficient neonatal donors improves engraftment and function. Am J Transplant. (2011) 11:2593–602. doi: 10.1111/j.1600-6143.2011.03720.x 21883917 PMC3226931

[49] Padler-Karavani V, Varki A. Potential impact of the non-human sialic acid N-glycolylneuraminic acid on transplant rejection risk. Xenotransplantation. (2011) 18:1–5. doi: 10.1111/j.1399-3089.2011.00622.x 21342282 PMC3098739

[50] Byrne G, Ahmad-Villiers S, Du Z, McGregor C. B4GALNT2 and xenotransplantation: a newly appreciated xenogeneic antigen. Xenotransplantation. (2018) 25:e12394. doi: 10.1111/xen.12394 29604134 PMC6158069

[51] Martens GR, Reyes LM, Li P, Butler JR, Ladowski JM, Estrada JL, Humoral reactivity of renal transplant-waitlisted patients to cells from GGTA1/CMAH/B4GalNT2, and SLA class I knockout pigs. Transplantation. (2017) 101:e86–92. doi: 10.1097/TP.0000000000001646 28114170 PMC7228580

[52] Salama A, Mosser M, Leveque X, Perota A, Judor JP, Danna C, Neu5Gc and alpha1–3 GAL xenoantigen knockout does not affect glycemia homeostasis and insulin secretion in pigs. Diabetes. (2017) 66:987–93. doi: 10.2337/db16-1060 28082457

[53] Hawthorne WJ, Salvaris EJ, Phillips P, Hawkes J, Liuwantara D, Burns H, Control of IBMIR in neonatal porcine islet xenotransplantation in baboons. Am J Transplant. (2014) 14:1300–9. doi: 10.1111/ajt.12722 24842781 PMC4204157

[54] Bottino R, Wijkstrom M, van der Windt DJ, Hara H, Ezzelarab M, Murase N, Pig-to-monkey islet xenotransplantation using multi-transgenic pigs. Am J Transplant. (2014) 14:2275–87. doi: 10.1111/ajt.12868 25220221 PMC4169326

[55] Hawthorne WJ, Salvaris EJ, Chew YV, Burns H, Hawkes J, Barlow H, Xenotransplantation of genetically modified neonatal pig islets cures diabetes in baboons. Front Immunol. (2022) 13:898948. doi: 10.3389/fimmu.2022.898948 35784286 PMC9243461

[56] Yamamoto T, Hara H, Foote J, Wang L, Li Q, Klein EC, Life-supporting kidney xenotransplantation from genetically engineered pigs in baboons: a comparison of two immunosuppressive regimens. Transplantation. (2019) 103:2090–104. doi: 10.1097/TP.0000000000002796 31283686

[57] Buhler L, Awwad M, Basker M, Gojo S, Watts A, Treter S, High-dose porcine hematopoietic cell transplantation combined with CD40 ligand blockade in baboons prevents an induced anti-pig humoral response. Transplantation. (2000) 69:2296–304. doi: 10.1097/00007890-200006150-00013 10868629

[58] Iwase H, Ekser B, Satyananda V, Bhama J, Hara H, Ezzelarab M, Pig-to-baboon heterotopic heart transplantation—exploratory preliminary experience with pigs transgenic for human thrombomodulin and comparison of three costimulation blockade-based regimens. Xenotransplantation. (2015) 22:211–20. doi: 10.1111/xen.12167 25847282 PMC4464944

[59] Cardona K, Korbutt GS, Milas Z, Lyon J, Cano J, Jiang W, Long-term survival of neonatal porcine islets in nonhuman primates by targeting costimulation pathways. Nat Med. (2006) 12:304–6. doi: 10.1038/nm1375 16501570

[60] Yoon IH, Chung H, Kim HJ, Nam HY, Shin JS, Kim YH, Peri-graft porcine-specific CD4(+) FoxP3(+) regulatory T cells by CD40-CD154 blockade prevented the rejection of porcine islet graft in diabetic mice. Xenotransplantation. (2019) 26:e12533. doi: 10.1111/xen.12533 31111577

[61] Kawai T, Andrews D, Colvin RB, Sachs DH, Cosimi AB. Thromboembolic complications after treatment with monoclonal antibody against CD40 ligand. Nat Med. (2000) 6:114. doi: 10.1038/72162 10655073

[62] Buhler L, Basker M, Alwayn IP, Goepfert C, Kitamura H, Kawai T, Coagulation and thrombotic disorders associated with pig organ and hematopoietic cell transplantation in nonhuman primates. Transplantation. (2000) 70:1323–31. doi: 10.1097/00007890-200011150-00010 11087147

[63] Boumpas DT, Furie R, Manzi S, Illei GG, Wallace DJ, Balow JE, A short course of BG9588 (anti-CD40 ligand antibody) improves serologic activity and decreases hematuria in patients with proliferative lupus glomerulonephritis. Arthritis Rheum. (2003) 48:719–27. doi: 10.1002/art.10856 12632425

[64] Shin JS, Kim JM, Kim JS, Min BH, Kim YH, Kim HJ, Long-term control of diabetes in immunosuppressed nonhuman primates (NHP) by the transplantation of adult porcine islets. Am J Transplant. (2015) 15:2837–50. doi: 10.1111/ajt.13345 26096041

[65] Shin JS, Kim JM, Min BH, Yoon IH, Kim HJ, Kim JS, Pre-clinical results in pig-to-non-human primate islet xenotransplantation using anti-CD40 antibody (2C10R4)-based immunosuppression. Xenotransplantation. (2018) 25. doi: 10.1111/xen.12356 PMC5809197 29057561

[66] Mohiuddin MM, Reichart B, Byrne GW, McGregor CGA. Current status of pig heart xenotransplantation. Int J Surg. (2015) 23:234–9. doi: 10.1016/j.ijsu.2015.08.038 26318967 PMC4684783

[67] Thompson P, Badell IR, Lowe M, Turner A, Cano J, Avila J, Alternative immunomodulatory strategies for xenotransplantation: CD40/154 pathway-sparing regimens promote xenograft survival. Am J Transplant. (2012) 12:1765–75. doi: 10.1111/j.1600-6143.2012.04031.x 22458586 PMC3387302

[68] Sakaguchi S, Miyara M, Costantino CM, Hafler DA. FOXP3+ regulatory T cells in the human immune system. Nat Rev Immunol. (2010) 10:490–500. doi: 10.1038/nri2785 20559327

[69] Chen D, Zhang N, Fu S, Schroppel B, Guo Q, Garin A, CD4+ CD25+ regulatory T-cells inhibit the islet innate immune response and promote islet engraftment. Diabetes. (2006) 55:1011–21. doi: 10.2337/diabetes.55.04.06.db05-1048 16567523

[70] Graca L, Le Moine A, Lin CY, Fairchild PJ, Cobbold SP, Waldmann H. Donor-specific transplantation tolerance: the paradoxical behavior of CD4+CD25+ T cells. Proc Natl Acad Sci USA. (2004) 101:10122–6. doi: 10.1073/pnas.0400084101 15218097 PMC454175

[71] Pathak S, Meyer EH. Tregs and mixed chimerism as approaches for tolerance induction in islet transplantation. Front Immunol. (2020) 11:612737. doi: 10.3389/fimmu.2020.612737 33658995 PMC7917336

[72] Yi S, Ji M, Wu J, Ma X, Phillips P, Hawthorne WJ, Adoptive transfer with in vitro expanded human regulatory T cells protects against porcine islet xenograft rejection via interleukin-10 in humanized mice. Diabetes. (2012) 61:1180–91. doi: 10.2337/db11-1306 22403295 PMC3331767

[73] Shin JS, Min BH, Kim JM, Kim JS, Yoon IH, Kim HJ, Failure of transplantation tolerance induction by autologous regulatory T cells in the pig-to-non-human primate islet xenotransplantation model. Xenotransplantation. (2016) 23:300–9. doi: 10.1111/xen.12246 27387829

[74] Sykes M Mixed chimerism and transplant tolerance. Immunity. (2001) 14:417–24. doi: 10.1016/S1074-7613(01)00122-4 11336687

[75] Kawai T, Sachs DH, Sykes M, Cosimi AB, Immune Tolerance N. HLA-mismatched renal transplantation without maintenance immunosuppression. N Engl J Med. (2013) 368:1850–2. doi: 10.1056/NEJMc1213779 23656665 PMC3760499

[76] Leventhal J, Abecassis M, Miller J, Gallon L, Tollerud D, Elliott MJ, Tolerance induction in HLA disparate living donor kidney transplantation by donor stem cell infusion: durable chimerism predicts outcome. Transplantation. (2013) 95:169–76. doi: 10.1097/TP.0b013e3182782fc1 23222893 PMC3531567

[77] Scandling JD, Busque S, Shizuru JA, Lowsky R, Hoppe R, Dejbakhsh-Jones S, Chimerism, graft survival, and withdrawal of immunosuppressive drugs in HLA matched and mismatched patients after living donor kidney and hematopoietic cell transplantation. Am J Transplant. (2015) 15:695–704. doi: 10.1111/ajt.13091 25693475

[78] Oura T, Ko DS, Boskovic S, O’Neil JJ, Chipashvili V, Koulmanda M, Kidney versus islet allograft survival after induction of mixed chimerism with combined donor bone marrow transplantation. Cell Transplant. (2016) 25:1331–41. doi: 10.3727/096368915X688966 26337731 PMC4946954

[79] Yang YG, deGoma E, Ohdan H, Bracy JL, Xu Y, Iacomini J, Tolerization of anti-Galalpha1–3Gal natural antibody-forming B cells by induction of mixed chimerism. J Exp Med. (1998) 187:1335–42. doi: 10.1084/jem.187.8.1335 9547344 PMC2212239

[80] Ohdan H, Yang YG, Shimizu A, Swenson KG, Sykes M. Mixed chimerism induced without lethal conditioning prevents T cell- and anti-Gal alpha 1,3Gal-mediated graft rejection. J Clin Invest. (1999) 104:281–90. doi: 10.1172/JCI6656 10430609 PMC408419

[81] Griesemer A, Liang F, Hirakata A, Hirsh E, Lo D, Okumi M, Occurrence of specific humoral non-responsiveness to swine antigens following administration of GalT-KO bone marrow to baboons. Xenotransplantation. (2010) 17:300–12. doi: 10.1111/j.1399-3089.2010.00600.x 20723202 PMC2942069

[82] Liang F, Wamala I, Scalea J, Tena A, Cormack T, Pratts S, Increased levels of anti-non-Gal IgG following pig-to-baboon bone marrow transplantation correlate with failure of engraftment. Xenotransplantation. (2013) 20:458–68. doi: 10.1111/xen.12065 24289469 PMC3848062

[83] Tena AA, Sachs DH, Mallard C, Yang YG, Tasaki M, Farkash E, Prolonged survival of pig skin on baboons after administration of pig cells expressing human CD47. Transplantation. (2017) 101:316–21. doi: 10.1097/TP.0000000000001267 27232934 PMC5124423

[84] Tasaki M, Wamala I, Tena A, Villani V, Sekijima M, Pathiraja V, High incidence of xenogenic bone marrow engraftment in pig-to-baboon intra-bone bone marrow transplantation. Am J Transplant. (2015) 15:974–83. doi: 10.1111/ajt.13070 25676635 PMC4407988

[85] Watanabe H, Ariyoshi Y, Pomposelli T, Takeuchi K, Ekanayake-Alper DK, Boyd LK, Intra-bone bone marrow transplantation from hCD47 transgenic pigs to baboons prolongs chimerism to >60 days and promotes increased porcine lung transplant survival. Xenotransplantation. (2020) 27:e12552. doi: 10.1111/xen.12552 31544995 PMC7007336

[86] Zhao Y, Swenson K, Sergio JJ, Arn JS, Sachs DH, Sykes M. Skin graft tolerance across a discordant xenogeneic barrier. Nat Med. (1996) 2:1211–6. doi: 10.1038/nm1196-1211 8898747

[87] Yamada K, Shimizu A, Ierino FL, Utsugi R, Barth RN, Esnaola N, Thymic transplantation in miniature swine. Development I, and function of the “thymokidney”. Transplantation. (1999) 68:1684–92. doi: 10.1097/00007890-199912150-00011 10609944

[88] LaMattina JC, Kumagai N, Barth RN, Yamamoto S, Kitamura H, Moran SG, Vascularized thymic lobe transplantation in miniature swine: I. Vascularized thymic lobe allografts support thymopoiesis. Transplantation. (2002) 73:826–31. doi: 10.1097/00007890-200203150-00032 11907438

[89] Yamada K, Shimizu A, Utsugi R, Ierino FL, Gargollo P, Haller GW, Thymic transplantation in miniature swine. II. Induction of tolerance by transplantation of composite thymokidneys to thymectomized recipients. J Immunol. (2000) 164:3079–86. doi: 10.4049/jimmunol.164.6.3079 10706697

[90] Yamada K, Vagefi PA, Utsugi R, Kitamura H, Barth RN, LaMattina JC, Thymic transplantation in miniature swine: III. Induction of tolerance by transplantation of composite thymokidneys across fully major histocompatibility complex-mismatched barriers. Transplantation. (2003) 76:530–6. doi: 10.1097/01.TP.0000080608.42480.E8 12923439

[91] Kamano C, Vagefi PA, Kumagai N, Yamamoto S, Barth RN, LaMattina JC, Vascularized thymic lobe transplantation in miniature swine: thymopoiesis and tolerance induction across fully MHC-mismatched barriers. Proc Natl Acad Sci USA. (2004) 101:3827–32. doi: 10.1073/pnas.0306666101 15007168 PMC374329

[92] Kumagai N, LaMattina JC, Kamano C, Vagefi PA, Barth RN, O’Neil JJ, Vascularized islet cell transplantation in miniature swine: islet-kidney allografts correct the diabetic hyperglycemia induced by total pancreatectomy. Diabetes. (2002) 51:3220–8. doi: 10.2337/diabetes.51.11.3220 12401713

[93] Kumagai N, O’Neil JJ, Barth RN, LaMattina JC, Utsugi R, Moran SG, Vascularized islet-cell transplantation in miniature swine. I. Preparation of vascularized islet kidneys. Transplantation. (2002) 74:1223–30. doi: 10.1097/00007890-200211150-00005 12451257

[94] Yamada K, Hirakata A, Tchipashvili V, Shimizu A, Iwaki H, Griesemer A, Composite islet-kidneys from single baboon donors cure diabetes across fully allogenic barriers. Am J Transplant. (2011) 11:2603–12. doi: 10.1111/j.1600-6143.2011.03733.x 21929644 PMC3226882

